# Parthenolide acts as a cocaine antagonist in the human dopamine transporter

**DOI:** 10.17912/micropub.biology.001720

**Published:** 2025-08-27

**Authors:** Oné R. Pagán

**Affiliations:** 1 Department of Biology, West Chester University, West Chester, Pennsylvania, United States

## Abstract

In this work, I describe the effects of parthenolide, a naturally occurring sesquiterpene lactone, on radiolabeled cocaine binding and on radiolabeled dopamine uptake in the human dopamine transporter expressed on HEK-293 cells (HEK/DAT cells). Parthenolide displaces radiolabeled cocaine in a concentration-dependent manner and does not display any significant inhibition of radiolabeled dopamine uptake. Further, parthenolide seems to alleviate the inhibition of dopamine uptake by 5 μM unlabeled cocaine at sub-toxic parthenolide concentrations.

**Figure 1. This figure depicts the compounds used in this work, the verification of the expression of dopamine transporter in HEK-293 cells, and the determination of the time to reach steady state and equilibrium binding of radiolabeled dopamine and radiolabeled cocaine, respectively. Additionally, the figure shows that parthenolide displaces radiolabeled cocaine binding without affecting radiolabeled dopamine uptake and alleviates unlabeled cocaine inhibition of dopamine uptake:**
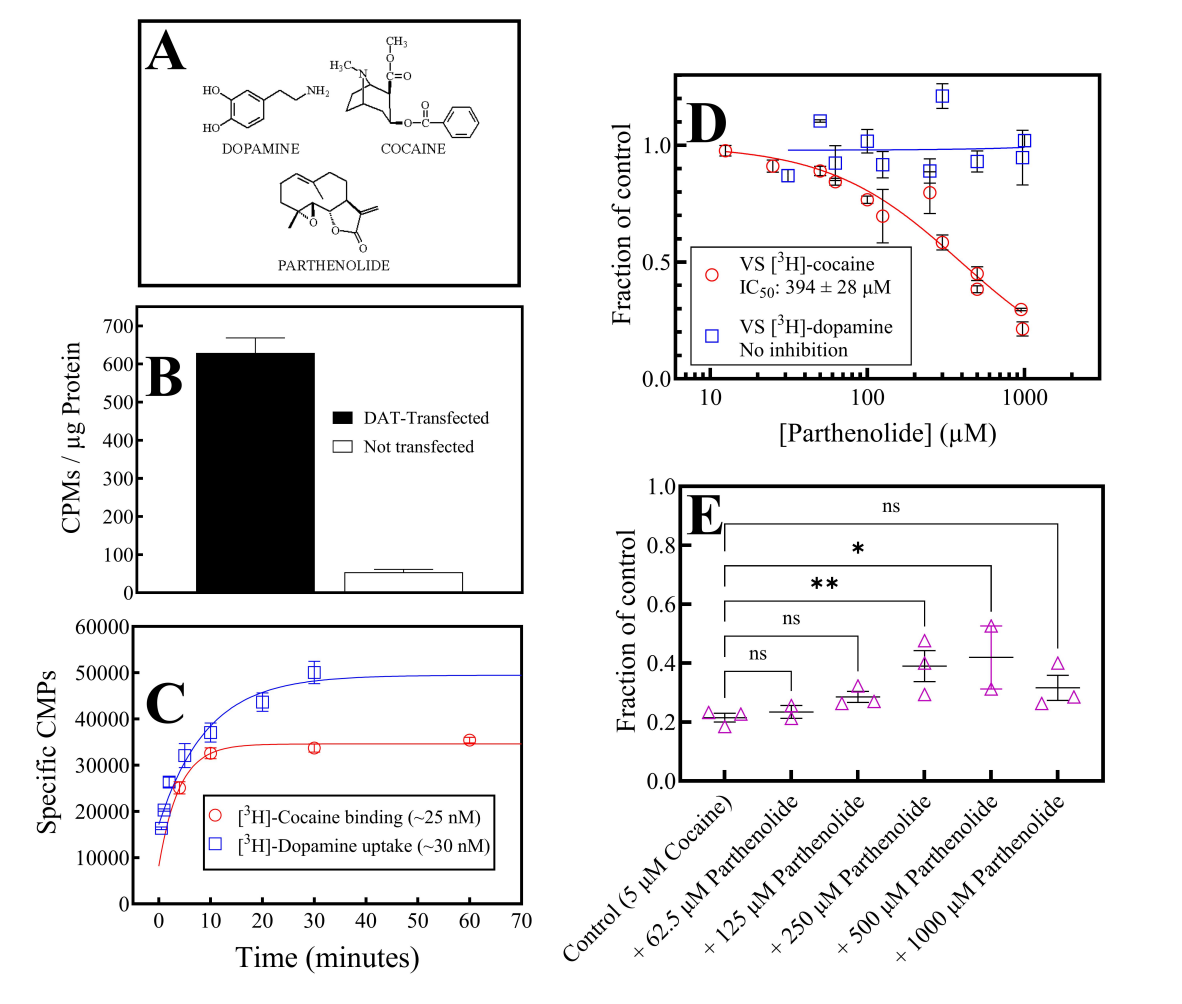
A: The chemical structures of dopamine, cocaine, and parthenolide, as indicated. B. [ ^3^ H]-cocaine (20 nM) binding to membranes from DAT-transfected or untransfected HEK-293 cells (N = 3-5). C. Time course curves for the binding of [ ^3^ H]-cocaine or [ ^3^ H]-dopamine uptake, as indicated (N = 3-4). D: Concentration-response plots of parthenolide vs. radiolabeled cocaine binding or radiolabeled dopamine uptake, as indicated. The data was analyzed using Equation 1 (N = 3). E: Parthenolide alleviates the inhibition of dopamine uptake by 5 μM cocaine at parthenolide concentrations of 250 and 500 μM (p = 0.0097 and 0.0166 respectively, by Dunnett’s multiple comparisons test; overall Two-way ANOVA: p = 0.0098; N = 2-3). The error bars in each graph (Figure 1 B to E) represent the standard error of the mean.

## Description

Parthenolide ( Figure 1A ) is a naturally occurring product belonging to the sesquiterpene lactone family of compounds. Parthenolide is mainly obtained from the Feverfew plant ( *Tanacetum parthenium* ) and is currently studied for its antitumoral and anti-inflammatory effects, among other applications (Liu et al., 2023). Neurotransmitter transporters are a ubiquitous class of proteins that are involved in neurotransmitter inactivation and are involved in many physiological and pathological states, including drug addiction (Ayala-López and Watts, 2021). Specifically, the human dopamine transporter is the main biochemical target of the abused drug cocaine (Izenwasser, 2004). Using the flatworm *planaria* as an animal model, my laboratory discovered that parthenolide alleviated cocaine’s acute and chronic behavioral effects in this organism (Pagán et al., 2008; Rowlands and Pagán, 2008; Baker et al., 2011; Pagán et al., 2012; Kakuturu et al., 2024; reviewed in Pagán 2014, 2017). Additionally, the laboratory of a collaborator, Dr. Carlos Jiménez-Rivera, discovered that parthenolide inhibits the cocaine-induced spontaneous firing activity of dopaminergic neurons in the ventral tegmental area of rats induced (Schwarz et al., 2011). The aforementioned body of parthenolide/cocaine work was inspired by original work described in Pagán (2005). In the present work, I describe the use of HEK-293 cells stably transfected with the human dopamine transporter (HEK/DAT cells) to explore the effects of parthenolide on cocaine binding, dopamine uptake, and the inhibition of dopamine uptake by cocaine.

Figure 1B shows a graph comparing the specific binding of 20 nM [ ^3^ H]-cocaine to either untransfected HEK-293 cells or HEK-293 cells stably transfected with the human dopamine transporter (HEK/DAT cells). Figure 1C shows time course graphs of either [ ^3^ H]-cocaine binding or [ ^3^ H]-dopamine uptake in HEK/DAT cells. Based on this data, all subsequent [ ^3^ H]-dopamine uptake or [ ^3^ H]-cocaine binding measurements were taken at 10 minutes, which corresponds to both equilibrium binding for cocaine or the steady-state rising phase of dopamine uptake. The HEK/DAT cells were further characterized by competition studies of ~25 nM [ ^3^ H]-cocaine against unlabeled cocaine, unlabeled dopamine, BTCP, and mazindol, all established ligands of the dopamine transporter (Appell et al., 2004; Tidjane Corera et al., 2001), with affinity values consistent with the scientific literature (Pagán, 2005).

Figure 1D shows concentration-response plots of parthenolide vs. [ ^3^ H]-cocaine binding using membranes from HEK/DAT cells or [ ^3^ H]-dopamine uptake using HEK/DAT cells in culture. Parthenolide inhibited cocaine binding with an IC _50_ of 394 ± 28 μM with no appreciable inhibition of dopamine uptake. The Hill coefficient value for the radiolabeled cocaine curve was 1.05 ± 0.09, suggesting a single class of sites for [ ^3^ H]-cocaine on the dopamine transporter. Parthenolide also alleviated the inhibition of dopamine uptake by 5 μM cocaine ( Figure 1E ). This cocaine concentration reduces dopamine uptake in these cells by about 80 % (Pagán 2005; “Control” in Figure 1E ). Parthenolide alleviates this 5 μM cocaine-induced decrease in dopamine uptake in two of the five tested concentrations (250 and 500 μM). This alleviation disappears at a parthenolide concentration of 1 mM ( Figure 1E ), likely due to parthenolide’s cytotoxicity (Freund et al., 2020). Taken together, at a first approximation, in addition to the experiments from my laboratory and collaborators using planarians and rats, the data shown in the present work further make the case for additional exploration of the parthenolide/cocaine interaction using various experimental models and approaches, with the assumption that parthenolide and cocaine interact through the dopamine transporter.

## Methods

General laboratory reagents and supplies were purchased from Fisher Scientific, Sigma-Aldrich, or Tocris. [ ^3^ H]-cocaine (46-50 Ci/mmol) was obtained from Perkin-Elmer. [ ^3^ H]-dopamine (50 Ci/mmol) was from Amersham (Piscataway, NJ). For this work, I used HEK-293 cells stably transfected with the wild-type human dopamine transporter. All statistical procedures and graphs were done with the Prism software package (GraphPad Software, San Diego, CA). Cells were cultured using Dulbecco’s modified Eagle medium F-12 with 10 % calf serum and PS (100 IU of penicillin plus 100 mg/L streptomycin; Ferrer and Javitch, 1998) and plated in 25 cm ^2^ flasks. When the cells were about 70-80 % confluent, they were suspended in culture media, and 800 μL aliquots of this suspension were added to individual collagen-type I-coated wells of a 24-well dish and grown to confluence (typically 3-4 days) before each experiment. The cells in each individual well were examined microscopically to verify that they were organized in a monolayer, reasonably ensuring that a similar number of cells were present in the wells when tested. All experiments were done at room temperature in phosphate-buffered saline solution (PBS) adjusted to pH 7.4, containing 100 µM tropolone (an antioxidant and an inhibitor of the enzyme catechol- *O* -methyl transferase; HEK-293 cells express this enzyme, which is part of dopamine metabolism (Eshleman et al., 1997). Radiolabeled dopamine uptake was done in HEK/DAT cells in culture, and radiolabeled cocaine binding was done using HEK/DAT membranes. All experiments were done in 1 % DMSO, a solubilizing agent due to parthenolide’s hydrophobicity. Benzo-tenocyclidine (BTCP) was used to stop dopamine uptake (1 mM) and to account for nonspecific binding or uptake (100 µM). BTCP is a dopamine reuptake blocker that binds in a mutually exclusive manner with cocaine in the human dopamine transporter (Tidjane Corera et al., 2001). The membrane preparation protocol was based on an established procedure (Houlihan et al., 2002).

**[ ^3^ H]-dopamine uptake.** A solution of [ ^3^ H]-dopamine was prepared to a final concentration of about 20-30 nM. This dopamine concentration is within the physiological range for vertebrates (Cragg and Rice, 2004). In a typical uptake experiment, there were three control wells (no parthenolide) and triplicate wells with several parthenolide concentrations. The culture medium from each well was removed, and each well was gently washed with 300 μL of PBS/ 100 uM tropolone. This wash was also discarded. Media containing the indicated [ ^3^ H]-dopamine concentration was added to each well and incubated for ten minutes. The reaction was stopped after 10 minutes by adding BTCP to a final concentration of 1 mM. The radiolabeled dopamine/BTCP solution was removed, and each well was washed with PBS/tropolone as above. A solution of 1 mM sodium citrate and 1 % Sodium dodecyl sulfate (SDS) was then added to each well, and the dish was shaken for 30 minutes at about 100 revolutions per minute (RPM). Three to four 10 μL aliquots of this solution were transferred to separate 20 mL scintillation vials containing 10 mL of scintillation cocktail (ScintiSafe 30 %, Fisher), shaken overnight, and counted using a Beckman LS-1801 scintillation counter.

**[ ^3^ H]-cocaine binding.** A solution of [ ^3^ H]-cocaine was prepared to a final concentration of ~ 25-30 nM. Briefly, membrane aliquots were mixed with buffer with or without parthenolide in separate 1.5 mL Eppendorf vials. Vials containing 100 μM BTCP were used to assess nonspecific binding. Upon incubation for 10 minutes, binding was stopped by vacuum filtration through glass fiber filters (GF-F, Whatman), followed by a 5 mL wash using ice-cold buffer, with a vacuum flow rate of 1 mL/second. Upon washing, each filter was transferred to a 20 mL scintillation vial containing ScintiSafe 30 % and counted as above. For both the reuptake and binding experiments, the data was analyzed using a modification of an empirical Hill equation (Weiss, 1997).

F = IC _50_^n^ / (IC _50_^n^ + P) [Equation 1]

F is the fraction of control of [ ^3^ H]-cocaine binding or [ ^3^ H]-dopamine uptake, P is the parthenolide concentration (μM), n is the Hill coefficient, and the IC _50_ is the parthenolide concentration inhibiting 50 % of [ ^3^ H]-cocaine binding or [ ^3^ H]-dopamine uptake.

**Alleviation experiments.** These experiments were done using the same procedures as in the [ ^3^ H]-dopamine uptake experiments described above. Briefly, the inhibition of [ ^3^ H]-dopamine uptake by 5 μM unlabeled cocaine was measured in the absence or in the presence of increasing concentrations of parthenolide.
